# Commentary: Effect of Angiotensin-Converting-Enzyme Inhibitor and Angiotensin II Receptor Antagonist Treatment on ACE2 Expression and SARS-CoV-2 Replication in Primary Airway Epithelial Cells

**DOI:** 10.3389/fphar.2022.842512

**Published:** 2022-01-28

**Authors:** Fedor Simko, Tomas Baka

**Affiliations:** ^1^ Institute of Pathophysiology, Faculty of Medicine, Comenius University, Bratislava, Slovakia; ^2^ 3rd Department of Internal Medicine, Faculty of Medicine, Comenius University, Bratislava, Slovakia; ^3^ Institute of Experimental Endocrinology, Biomedical Research Center, Slovak Academy of Sciences, Bratislava, Slovakia

**Keywords:** angiotensin II, angiotensin 1–7, ACE2, COVID-19, SARS-CoV-2, captopril, losartan

**A Commentary on**

**Effect of Angiotensin-Converting-Enzyme Inhibitor and Angiotensin II Receptor Antagonist Treatment on ACE2 Expression and SARS-CoV-2 Replication in Primary Airway Epithelial Cells**

*by Okoloko, O., Vanderwall, E. R., Rich, L. M., White, M. P., Reeves, S. R., Harrington, W. E., et al. (2021). Front. Pharmacol. 12, 765951. doi:* *10.3389/fphar.2021.765951*

## Introduction

Curbing activation of the renin-angiotensin-aldosterone system (RAAS) at various levels is considered the most effective protection in a number of cardiovascular pathologies. Two years of the Coronavirus disease 2019 (COVID-19) pandemic have revealed that therapeutic interference with the RAAS may be a much more complex issue than ever expected. During an acute stress reaction, the RAAS is the crucial neurohumoral actor covering high circulatory and metabolic demands. On the other hand, long-lasting stimulation of the RAAS in the course of chronic cardiovascular disorders may result in undesirable target organ rebuilding and energy depletion, thus compromising cardiovascular health and life expectancy (Simko and Simko, 1999). The alternative angiotensin-converting enzyme (ACE)2/angiotensin (Ang) 1–7/Mas receptor (MasR) pathway counterbalances the potentially harmful effects of the classical ACE/AngII/angiotensin II type 1 receptor (AT1R) pathway; hence, well balanced cooperation between these two pathways preserves the prevalence of benefits over the potential harm (Steckelings and Sumners, 2020; Simko et al., 2021).

## The Potential Ambivalent Impact of RAS-Inhibition in ACE2—COVID-19 Interactions

Besides their haemodynamic actions, both RAS pathways participate in the regulation of inflammatory reactions. As ACE2 has been shown to be the receptor and entrance route for severe acute respiratory syndrome coronavirus (SARS-CoV), its role in the SARS-CoV-2 pandemic has been addressed. Since older patients with cardiovascular pathologies, frequently treated with ACE inhibitors (ACEI) or AT1R blockers (ARB), suffer from a serious course of COVID-19 and high mortality, and RAS inhibitors seem to increase membrane-bound ACE2 (mACE2) expression, presumably thus facilitating the entry of SARS-CoV-2 into cells, it has been suggested that treatment with ACEI/ARB could increase the potential danger during the COVID-19 pandemic (Fang et al., 2020). On the other hand, the opposite view has been raised, that increased cellular ACE2 expression might reduce the AngII level by converting it to Ang1-7/Ang1-9, with a protective haemodynamic and anti-inflammatory impact. Moreover, the potential withdrawal of RAS inhibitors in cardiovascular patients could result in haemodynamic or atherosclerosis plaque destabilization and a surge in cardiovascular events (Vaduganathan et al., 2020). The results of several retrospective analyses and prospective clinical trials have not indicated any danger in terms of increased sensitivity to or seriousness of SARS-CoV-2 infection in the population treated with ACEI or ARB (Morales et al., 2021; Rothlin et al., 2021). In line with these findings, national cardiac societies recommended the continuation of ACEI/ARB therapies (Hrenak and Simko, 2020; Morales et al., 2021; Rothlin et al., 2021). However, studies elucidating the molecular effects of RAS inhibitors on the cellular level are lacking.

## Effect of Captopril/Losartan on ACE2 and SARS-CoV-2 Replication in Cultured Airway Epithelial Cells

Okoloko et al., in a paper recently published in *Frontiers in Pharmacology* (Okoloko et al., 2021), investigated whether captopril or losartan were able to modulate the expression of ACE2 in airway epithelial cell cultures from children or adults, as well as SARS-CoV-2 replication, or the expression of inflammatory mediators. The experiments showed that neither captopril nor losartan modified membrane-bound ACE2 (mACE2) expression in respiratory epithelial cell cultures, nor did these RAS inhibitors increase SARS-CoV-2 replication and the level of inflammatory cytokines or interferon type I/III in epithelial cell cultures exposed to SARS-CoV-2 compared to a SARS-CoV-2-treated cell line without RAS-inhibition. However, treatment with captopril/losartan, significantly increased mACE2 expression when compared to untreated control epithelial cell cultures (without SARS-CoV-2 or RAS inhibitors), a fact that remained unexplained by the authors (Okoloko et al., 2021).

## Discussion and Future Research Perspectives

It should be said that considerations on ACEI/ARB may cover different standpoints reflecting the complex nature and biological impact of the RAAS and its pharmacological modulation (Simko and Baka, 2021; Simko et al., 2021). The principal potential danger of the SARS-CoV-2—cell membrane interaction is, besides entry of the virus, the reduction of mACE2 expression due to internalization of the ACE2/SARS-CoV complex into the intracellular space and mACE2 shedding by the ACE2/SARS-CoV-complex-activated sheddase ADAM17 (tumour necrosis factor-α-converting enzyme) (Gheblawi et al., 2020; Oudit and Pfeffer, 2020). As a result, the proinflammatory action of AngII starts to dominate over the anti-inflammatory effects of the ACE2/Ang1-7/MasR pathway, thus potentiating oxidative stress, inflammation and even cytokine storm development (Hrenak and Simko, 2020). A cytokine storm represents an exaggerated inflammation characterized by a cytokine burst, resulting in severe multiorgan damage with a potentially fatal outcome (Mehta et al., 2020). A severe course of COVID-19 was shown to be associated with high plasma levels of interleukins 2, 7 and 10, granulocyte-colony stimulating factor, inducible protein 10, macrophage inflammatory protein 1-α, and tumour necrosis factor-α, resulting in pneumonia, acute respiratory distress syndrome (ARDS) or cardiac injury (Huang et al., 2020).

Hypothetically, SARS-CoV-2-induced reduction of mACE2 expression may be the virus’s self-protecting reaction against other viruses competing with SARS-CoV-2 for the ACE2-receptor to enter the cell. The high affinity of the SARS-CoV-2 spike protein to ACE2 potentially enables the cellular invasion and replication of this particular virus regardless of the number of mACE2 receptors. In line with the above presumption, it seems plausible that the reduction of mACE2 expression (by SARS-CoV-2 infection) rather than its enhancement (by ACEI/ARB or any other means) could be prognostically unfavourable in COVID-19 patients due to attenuation of the anti-inflammatory effect of the ACE2/Ang1-7/MasR and ACE2/alamandine/MrgD (Mas-related G-protein-coupled receptor D) pathways. Indeed, Pedrosa et al. have recently shown that candesartan and captopril upregulated the expression of ACE2, MasR and AngII type 2 receptor (AT2R) in the lungs of young rats or aged rats and rats with metabolic syndrome in *in vivo* experiments. In a cell culture of pneumocytes the SARS-CoV-2-spike protein led to a reduction of mACE2 expression by its cellular internalization and shedding via the enhancement of ADAM17 enzymatic activity. While mACE2 expression was reduced, the levels of internalized ACE2 in pneumocytes and soluble ACE2 (sACE2) in culture medium increased. Both captopril and candesartan increased mACE2 in rat lungs, apparently by inhibiting spike protein-internalization and by hampering spike-protein-ACE2 complex-induced activation of ADAM17, leading to blunted ACE2 shedding (Pedrosa et al., 2021; Simko and Baka, 2021).

Although in both studies, ACEI/ARB increased the mACE2 expression in controls, only Pedrosa et al. observed ACEI/ARB-induced mACE2 enhancement in spike-protein-exposed tissues both *in vivo* and *in vitro*, while Okoloko et al. did not show any change in ACE2 expression by RAS-inhibition in SARS-CoV-2-incubated airway epithelial cells. The reason underlying this difference is unclear but may be associated with the different design of the experiments, and the cells investigated. Since ACE2 is expressed differently within different organs and even in different cells of the same tissue, the use of distinct cell lines by Okoloko et al. and Pedrosa et al. may be the reason for some of the differences in their findings. Moreover, the binding of SARS-CoV-2 to ACE2 is determined by S-protein priming via the transmembrane protease serine 2 (TMPRSS2) (Hoffmann et al., 2020). Unfortunately, TMPRSS2, which may have influenced SARS-CoV-2 entry into both untreated or RAS inhibitor-treated cells, was not investigated in either of the mentioned experiments. Of note, the tropism of SARS-CoV-2 varies considerably for different species, with high tropism for humans but low tropism for rats or chickens, which may limit the translation of results achieved with rats into a clinical setting. Potentially, laboratory animals with high permissivity of ACE2 proteins to SARS-CoV-2, such as rabbits, cats or dogs (Conceicao et al., 2020) could yield different results.

Nevertheless, the principal message of both papers is similar, suggesting the apparent clinical safety of ACEI/ARB in COVID-19 patients (Okoloko et al., 2021; Pedrosa et al., 2021).

However, several matters remain to be investigated further:

• First, in both studies ACEI captopril and ARB candesartan/losartan yielded very similar results. Besides the reduction of AngII production, ACEI are known to increase the level of bradykinin with a potentially proinflammatory nature. On the other hand, ARB not only reduce AngII effects but increase the level of AT1R-unbound AngII molecules, potentially enabling their interaction with AT2R (Simko et al., 2003). This pathway may not only exert anti-inflammatory and antiproliferative action itself but may also support the mutual potentiation of the AngII/AT2R and the Ang1-7/MasR protective routes (Steckelings and Sumners, 2020). These differences between ACEI and ARB should not be ignored, as they may be of clinical importance.

• Second, the role of sACE2 remains to be more thoroughly elucidated. On the one hand, it may activate the plasma ACE2/Ang1-7/alamandine pathway, and more importantly it seems to represent a decoy for circulating SARS-CoV-2 virus, preventing its interactions with mACE2 and cellular internalization (Batlle et al., 2020); both actions are potentially protective. On the other hand, increased sACE2 could indicate accelerated mACE2 splitting, attenuating activation of the tissue ACE2/Ang1-7/MasR pathway, and increased sACE2 has been suggested as a potential marker of compromised prognosis in cardiovascular pathologies (Sama et al., 2020; Oudit and Pfeffer, 2020) or in patients with severe COVID-19 (Akin et al., 2021).

• Third, the inflammatory reaction against viruses is a phylogenetically established adaptive response to conquer an invader. Accordingly, increased AngII with cytokine activation is supposedly desirable to localize and damage the microorganisms in the initial phase of infection. The ACE2/Ang1-7/MasR or ACE2/alamandine/MrgD pathway should potentially dominate in the later phase of acute infection to calm the inflammation down and support reparative processes (Hrenak and Simko, 2020; Simko and Baka, 2021). Given that acute infection usually takes about 10–14 days, it does not seem unreasonable to suppose that patients not taking ACEI/ARB at the onset of SARS-CoV-2 infection could benefit from therapeutic introduction of RAS-inhibition in the later phase of infection. However, the complexity of the RAAS in the pathogenesis of cardiovascular pathologies or COVID-19 warns that RAS-inhibition should be done with considerable care.

**FIGURE 1:**
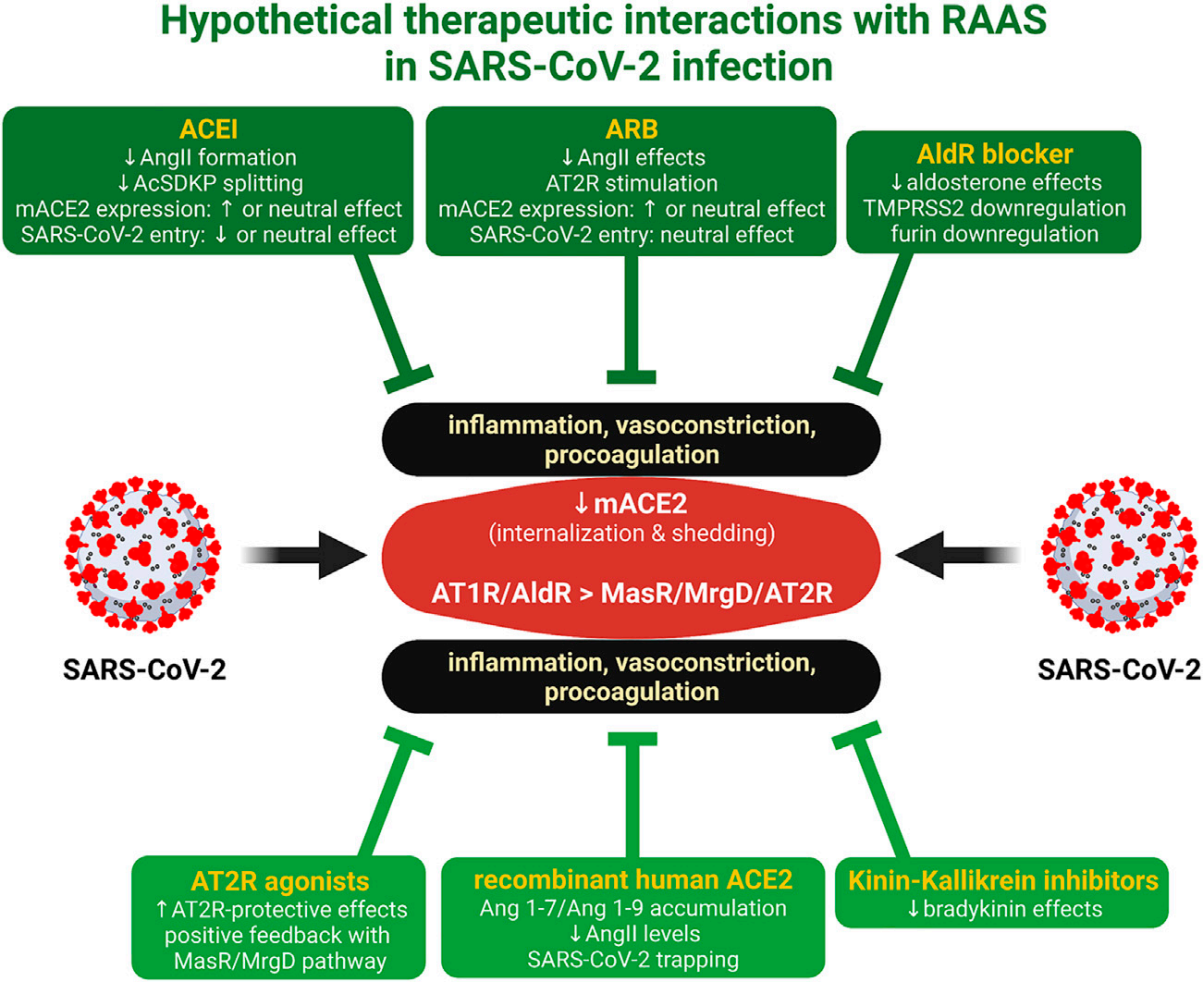
Hypothetical therapeutic interactions with RAAS in SARS-CoV-2 infection. ACE2 is the receptor and the entrance rout for SARS-CoV-2. The virus reduces the expression of membrane-bound ACE2 (mACE2) by internalization and shedding, resulting in the relative dominance of AT1 receptor (AT1R)/aldosterone receptor (AldR) deleterious effects over the Mas receptor (MasR)/MrgD alamandin receptor (MrgD)/angiotenin II type 2 receptor (AT2R) protective cardiovascular and anti-inflammatory actions. ACE inhibitors (ACEI), angiotensin II type 1 receptor blockers (ARB), aldosterone receptor (AldR) blockers (Wilcox and Pitt, 2020), AT2R agonists (Tornling et al., 2021), recombinant human ACE2 (Zhang et al., 2021), or kinin-kallikrein system inhibitors (Mansour et al., 2021) induce interactions with RAAS, which may render protection. Created with BioRender.com.

The fine-tuning of the ACEI/ARB regime and other RAS-associated neurohumoral cascade modulators, such as aldosterone antagonists, recombinant human ACE2, kinin-kallikrein inhibitors, or AT2R agonists, may result in the benefits outweighing the potential harm, thus making therapeutic RAAS modulation a hopeful means of fighting the COVID-19 pandemic (Figure 1).
